# Protection of Male Rat Offspring against Hypertension Programmed by Prenatal Dexamethasone Administration and Postnatal High-Fat Diet with the Nrf2 Activator Dimethyl Fumarate during Pregnancy

**DOI:** 10.3390/ijms20163957

**Published:** 2019-08-14

**Authors:** Chien-Ning Hsu, Yu-Ju Lin, Hong-Ren Yu, I-Chun Lin, Jiunn-Ming Sheen, Li-Tung Huang, You-Lin Tain

**Affiliations:** 1 Department of Pharmacy, Kaohsiung Chang Gung Memorial Hospital, Kaohsiung 833, Taiwan; 2 Department of Obstetrics and Gynecology, Kaohsiung Chang Gung Memorial Hospital and Chang Gung University College of Medicine, Kaohsiung 833, Taiwan; 3 Department of Pediatrics, Kaohsiung Chang Gung Memorial Hospital and Chang Gung University College of Medicine, Kaohsiung 833, Taiwan; 4 Department of Pediatrics, Chiayi Chang Gung Memorial Hospital and Chang Gung University College of Medicine, Kaohsiung 833, Taiwan; 5 Department of Traditional Chinese Medicine, Chang Gung University, Linkow 244, Taiwan

**Keywords:** asymmetric dimethylarginine, developmental origins of health and disease (DOHaD), hypertension, nuclear factor erythroid-derived 2-related factor 2 (Nrf2), nitric oxide, nutrient sensing signal, oxidative stress, renin-angiotensin system

## Abstract

Hypertension can originate from early-life exposure to oxidative stress. As reported, dimethyl fumarate (DMF) activates nuclear factor erythroid-derived 2-related factor 2 (Nrf2) and protects against oxidative stress damage. We examined whether maternal DMF therapy protects adult offspring against hypertension programmed by prenatal dexamethasone (DEX) and postnatal high-fat (HF) diet exposure. We examined male Sprague Dawley rat offspring at 4 months of age from five groups (*n* = 11–13/group): control, DEX (0.1mg/kg i.p. from gestational day 16 to 22), HF (D12331 diet from weaning to 16 weeks of age), DEX+HF, and DEX+HF+DMF (50mg/kg/day via gastric gavage for 3 weeks during pregnancy). Maternal DMF therapy prevented male offspring against hypertension programmed by combined DEX and HF exposures. The protective effects of maternal DMF include reduced oxidative stress, decreased plasma asymmetric dimethylarginine (ADMA) levels, downregulated the renin-angiotensin system (i.e. *Ren*, *Agt*, *Ace*, and *Agtr1a*), increased renal protein levels of certain nutrient-sensing signals, and promoted autophagy. In conclusion, maternal Nrf2 activation by DMF protects male adult offspring against hypertension programmed by combined DEX and HF exposures. Our results cast a new light on the therapeutic potential of targeting Nrf2 signaling pathway as reprogramming strategies to prevent programmed hypertension in children exposed to antenatal corticosteroids and postnatally excessive consumption of fat.

## 1. Introduction

Hypertension remains the major preventable cause of all-cause death globally, despite advanced drug treatment strategies. Adverse environments on pregnancy increase risk for developing hypertension in later life by so-called the developmental origins of health and diseases (DOHaD) [1]. The developing fetus is highly sensitive to oxidant injury because of its low antioxidant capacity [2]. Oxidative stress is an imbalance between pro-oxidant molecules and antioxidant defenses, mainly linked to dysregulation of reactive oxygen species (ROS) and nitric oxide (NO). There exists a considerable body of literature supporting early-life NO–ROS imbalance-mediated oxidative stress is capable of programming adult hypertension [3,4,5].

Although glucocorticoid administration is commonly used in premature infants suffering from respiratory distress syndrome to prevent chronic lung disease, major concern has been arisen about its long-term adverse effects [6]. We and others have shown that oxidative stress contributes to the development of hypertension programmed by antenatal glucocorticoid exposure [7,8]. Additionally, we previously reported that prenatal dexamethasone (DEX) administration induces programmed hypertension in adult offspring, which is exacerbated by postnatal high-fat (HF) diet [9,10]. Given that the close link between high-fat intake and oxidative stress in hypertension, these findings support the notion that postnatal factors (i.e. high-fat diet) could act as a “second hit” to deteriorate earlier programming mechanism (i.e., oxidative stress) induced by first hit (i.e., DEX exposure).

Blood pressure (BP) is tightly controlled by a complicated process that comprises major contributions from the kidney. Accordingly, renal programming is considered as a key mechanism of programmed hypertension [11]. We and others have identified several common mechanisms underpinning renal programming and hypertension programmed by glucocorticoid [12,13,14]. These mechanisms consist of oxidative stress, impaired nitric oxide (NO) pathway, dysregulated nutrient-sensing signaling, autophagy, and the inappropriate activation of the renin-angiotensin system (RAS).

Nuclear factor erythroid-derived 2-related factor 2 (Nrf2), a redox-sensing transcription factor, controls an array of antioxidant response element-dependent genes involved in the oxidative stress response [15,16]. Nrf2 activation has been shown to prevent hypertension in spontaneously hypertensive rats (SHRs) [17]. Additionally, Nrf2 activation is relevant to reserving the adverse programming effects in programmed hypertension models [18,19]. Dimethyl fumarate (DMF), a Nrf2 pathway activating agent, has been tested in human disorders, including multiple sclerosis [20] and psoriasis [21]. During organogenesis, oral administration of DMF to pregnant animals and women had no adverse effect on fetal development [22]. Additionally, a previous report demonstrated that DMF activates Nrf2 and thus protects the kidney against oxidative stress damage [23]. However, the reprogramming effects of maternal DMF treatment in programmed hypertension have rarely been studied.

The current study was therefore designed to examine whether maternal DMF therapy protects adult offspring against DEX+HF induced hypertension related to reduction of oxidative stress, blockade of the RAS, and rebalancing of the nutrient-sensing signals.

## 2. Results

We observed that male pup mortality rates, body weight, and kidney weight did not differ between the five groups analyzed (Table 1). The systolic and diastolic BPs, and mean arterial pressure (MAP) of DEX group were significantly higher than those in the control group. Similarly, postnatal HF diet caused a marked increase in BPs. The DEX+HF group had the highest BPs compared to DEX, HF, and control groups.

**Figure 1:**
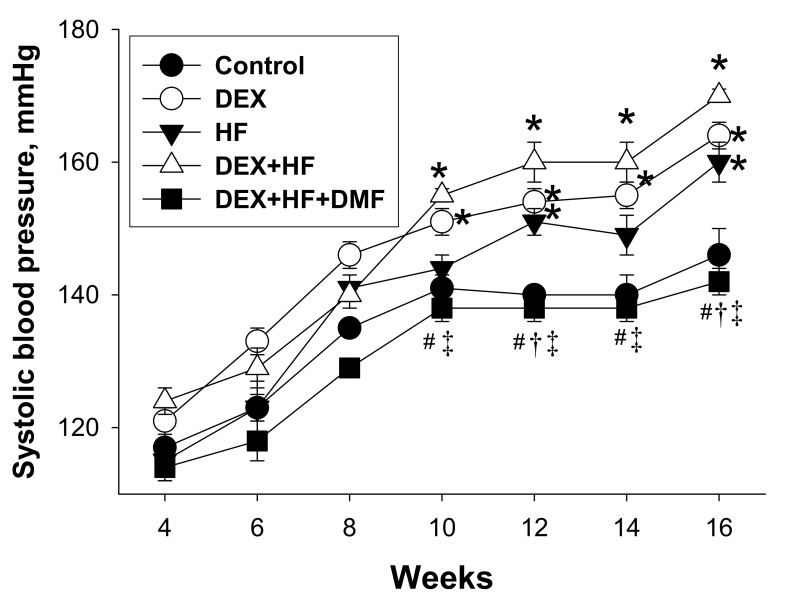
Effects of prenatal dexamethasone (DEX), postnatal high-fat diet (HF), and dimethyl fumarate (DMF) on systolic blood pressure in male offspring. * *p* < 0.05 vs. control; ^#^ *p* < 0.05 vs. DEX; ^†^ *p* < 0.05 vs. HF; ^‡^ *p* < 0.05 DEX+HF. *n* = 11–13/group.

Figure 1 shows that the elevation of SBP (~30 mmHg) in the DEX+HF group was starting from 10 to 16 weeks of age compared to controls. The reduction in SBP caused by maternal DMF treatment was significant for measurements taken at 10 to 16 weeks of age in the DEX+HF+DMF group vs. DEX+HF group, but not earlier. These findings showed that maternal DMF therapy prevented the synergistic interaction between the effects of prenatal DEX and postnatal HF on the development of hypertension.

**Figure 2:**
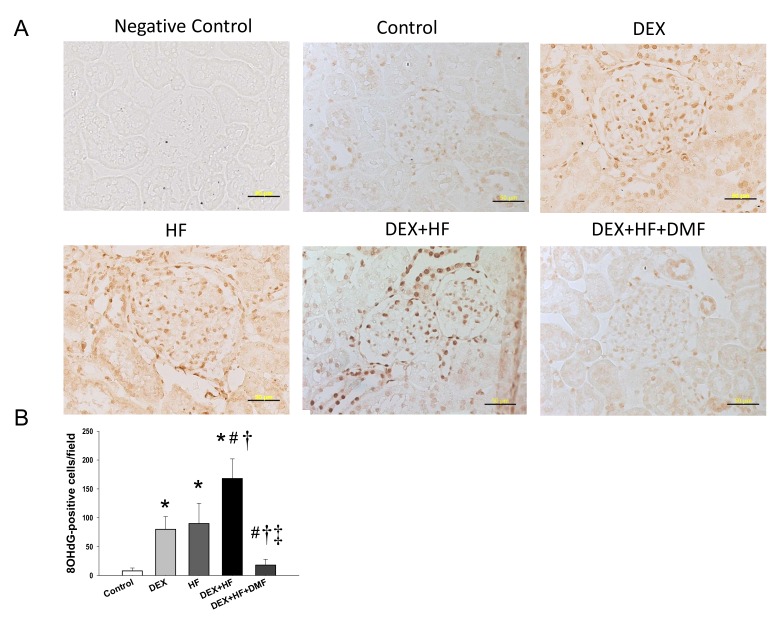
Immunohistochemical staining of 8-hydroxydeoxyguanosine (8-OHdG) at 16-week-old male offspring kidney. (**A**) Light micrographs illustrating immunostaining for 8-OHdG in the kidney exposed to prenatal dexamethasone (DEX), postnatal high-fat diet (HF), and dimethyl fumarate (DMF). Bar = 50 μm. (**B**) Quantitative analysis of 8-OHdG-positive cells per microscopic field (×400). * *p* < 0.05 vs. control; ^#^ *p* < 0.05 vs. DEX; ^†^ *p* < 0.05 vs. HF; ^‡^ *p* < 0.05 DEX+HF. *n* = 8/group.

Increased oxidative stress plays a role in programmed hypertension [5]. We first analyzed 8-hydroxydeoxyguanosine (8-OHdG), an indicator of oxidative stress related DNA damage, in offspring’s kidneys (Figure 2A). Immunostaining of 8-OHdG showed little staining in the glomeruli and renal tubules of the control group (8 ± 5 positive cells), an intermediate level of staining in the DEX (80 ± 22 positive cells) and HF (90 ± 35 positive cells) groups, and intense staining in the DEX+HF group (168 ± 34 positive cells) (Figure 2B). Of note, maternal DMF therapy restored the increase of renal 8-OHdG staining induced by DEX+HF exposure (18 ± 10 positive cells) (Figure 2B).

The link between oxidative stress and NO deficiency in programmed hypertension has been recognized [3,24]. NO deficiency can be attributed to decreased L-arginine (the substrate for NO synthase) and increased endogenous NOS inhibitors, asymmetric dimethylarginine (ADMA), and symmetric dimethylarginine (SDMA) [25]. We, hence, further investigated these components of NO pathway. As shown in Table 2, plasma level of L-citrulline (the precursor of l-arginine) was lower in the DEX+HF+DMF group compared to the DEX, HF, or DEX+HF group. Plasma levels of L-arginine and SDMA were not different among the five groups. Nevertheless, maternal DMF therapy significantly decreased ADMA level and increased l-arginine-to-ADMA ratio in the DEX+HF+DMF group vs. DEX+HF and HF group. As ADMA and l-arginine compete for NOS, the l-arginine-to-ADMA ratio has been used to represent NO bioavailability [26]. Our data indicate that the protective effects of maternal DMF therapy against programmed hypertension are, at least in part, due to decreased oxidative stress and restoration of ADMA-NO imbalance. 

**Figure 3:**
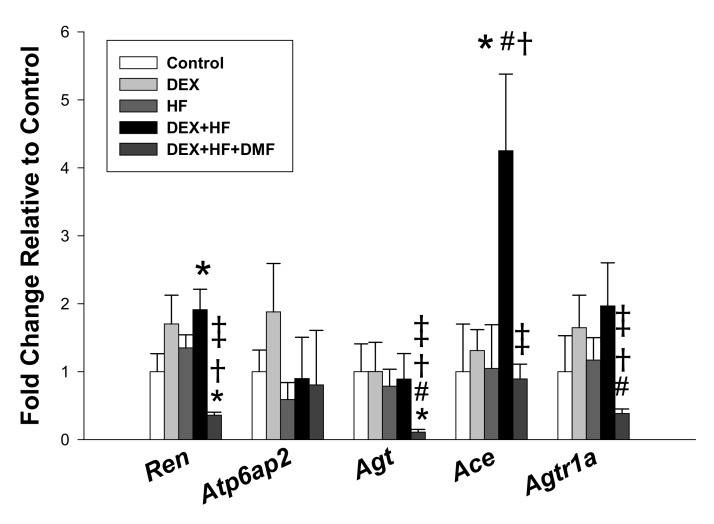
Effects of prenatal dexamethasone (DEX), postnatal high-fat diet (HF), and dimethyl fumarate (DMF) on mRNA expression of the renin-angiotensin system in male offspring kidneys at 16 weeks of age. * *p* < 0.05 vs. control; ^#^ *p* < 0.05 vs. DEX; ^†^ *p* < 0.05 vs. HF; ^‡^ *p* < 0.05 DEX+HF. *n* = 8/group.

As RAS activation is involved in hypertension programmed by DEX exposure [7,13], we next looked at the renal mRNA expression of RAS components. Renal mRNA expression of *Ren* and *Ace* were higher in DEX+HF group than in those of controls (Figure 3). Maternal DMF therapy reduced renal mRNA expression of *Ren*, *Agt*, *Ace*, and *Agtr1a* in the DEX+HF+DMF group vs. the DEX+HF group. Because activating angiotensin converting enzyme (ACE)-angiotensin (Ang) II-angiotensin type 1 receptor (AT1R) axis promotes vasoconstriction in favor of hypertension, our data provide a potential mechanism for the protective effect of DMF on DEX+HF-induced hypertension is, at least in part, due to blockade of the RAS.

**Figure 4:**
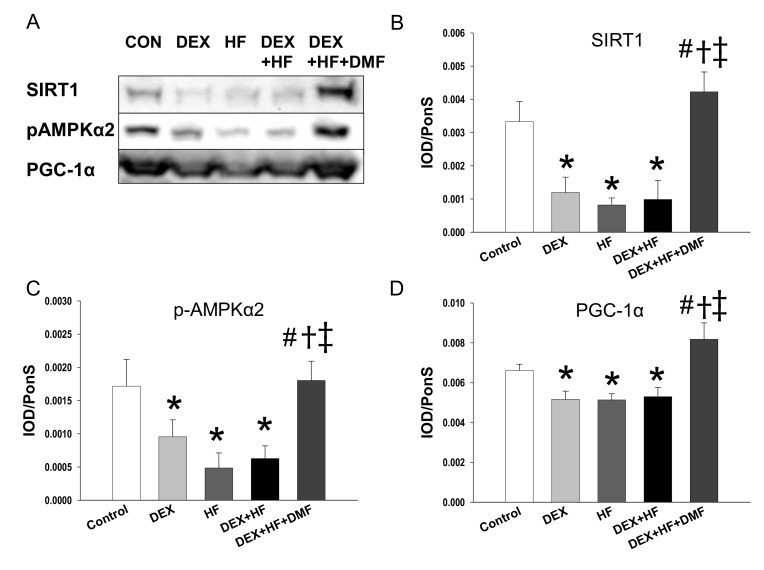
Protein levels of nutrient-sensing signals at 16-week-old offspring kidney exposed to prenatal dexamethasone (DEX), postnatal high-fat diet (HF), and dimethyl fumarate (DMF). (**A**) Presentative western blots of SIRT1 (120 kDa), phosphorylated AMPKα2 (62 kDa), and PGC-1α (90 kDa). Relative abundance of renal cortical (**B**) SIRT1, (**C**) p-mTOR, and (**D**) PGC-1α as quantified. *n* = 8/group. * *p* < 0.05 vs. control; ^#^ *p* < 0.05 vs. DEX; ^†^ *p* < 0.05 vs. HF; ^‡^ *p* < 0.05 DEX+HF.

Given that imbalanced metabolic status during pregnancy can disturb nutrient-sensing signals resulting in renal programming and developmental hypertension [5,27], we further analyzed nutrient-sensing signals (Figure 4). Several well-known nutrient-sensing signals are present in the kidney, including cyclic adenosine monophosphate (AMP)-activated protein kinase (AMPK), silent information regulator transcript (SIRT), peroxisome proliferator-activated receptors (PPARs), and PPARγ coactivator-1α (PGC-1α) [28]. We observed that renal protein levels of SIRT1 (Figure 4B), phosphorylated AMPKα2 (Figure 4C), and PGC-1α (Figure 4D) were decreased in the DEX, HF, and DEX+HF group vs. controls. While these changes were restored by maternal DMF therapy.

**Figure 5:**
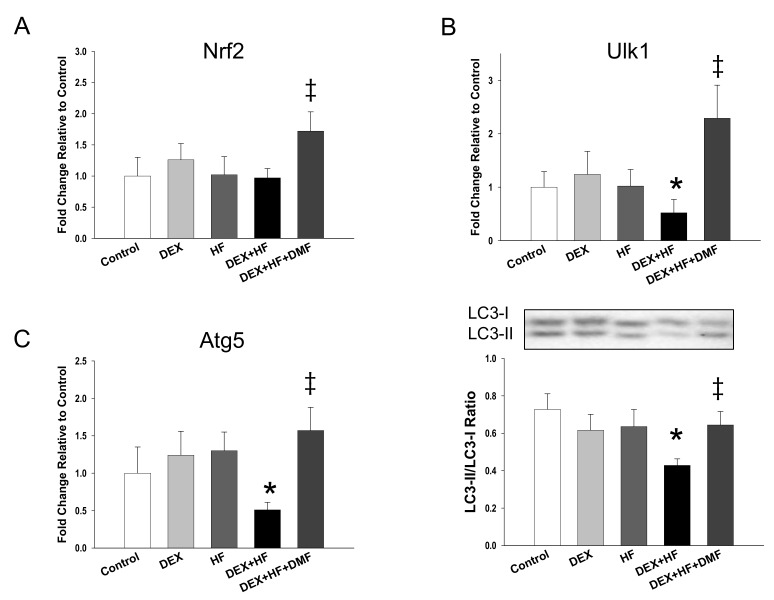
Effects of prenatal dexamethasone (DEX), postnatal high-fat diet (HF), and dimethyl fumarate (DMF) on mRNA expression of (**A**) *Nrf2*, (**B**) *Ulk1*, and (**C**) *Atg5*, and (**D**) protein levels of LC3-II (14 kDa)/LC3-I (16 kDa) ra tio in male offspring kidneys at 16 weeks of age. * *p* < 0.05 vs. control; ^‡^ *p* < 0.05 DEX+HF. *n* = 8/group.

Given that Nrf2 and PGC-1α are involved in autophagy [29,30], and that dysfunction of autophagy may lead to abnormal mitochondrial function and increase oxidative stress, we next examined whether autophagy in the kidneys is altered by DEX, HF intake, and DMF (Figure 5). Renal *Nrf2* mRNA expression was not different between the control, DEX, HF, and DEX+HF groups (Figure 5A). However, DMF therapy increased *Nrf2* mRNA expression compared to the DEX+HF group. We observed that DEX+HF decreased mRNA expression of *Ulk1* (Figure 5B) and *Atg5* (Figure 5C). Conversely, DMF treatment significantly increased renal mRNA expression of *Nrf2*, *Ulk1*, and *Atg5* compared to the DEX+HF group. A similar pattern of results was obtained in the LC3-II/LC3-I ratio, which was higher in the DEX+HF+DMF vs. DEX+HF group (Figure 5D). These results indicate that autophagy is inhibited by DEX+HF exposure, whereas it is promoted by DMF therapy.

## 3. Discussion

This study provides insight into the interactions of Nrf2, oxidative stress, nutrient-sensing signals, autophagy, and the RAS in the kidney by which maternal DMF therapy prevents hypertension programmed by prenatal DEX administration and post-weaning HF consumption in adult male offspring. Our major findings in the current study are (1) combined prenatal DEX administration and postnatal HF diet induced hypertension in adult male offspring, which maternal DMF therapy prevented; (2) DEX+HF two-hit-induced hypertension relates to increased oxidative stress, decreased NO bioavailability, activation of the RAS, downregulation of nutrient-sensing signals, and impaired autophagy; and (3) the beneficial effects of maternal DMF on programmed hypertension are associated with reduced oxidative stress, decreased plasma ADMA levels, increased plasma l-arginine-to-ADMA ratio, decreased renal mRNA of *Ren*, *Agt*, *Ace*, and *Agtr1a,* increased renal protein levels of SIRT1, phosphorylated AMPKα2, and PGC-1α, as well as promoted autophagy. 

The present report is consistent with the results of previous works showing that combined DEX and HF exposures synergistically increased BP in adult offspring [9,10]. Our data showed that maternal DMF treatment had no adverse effect on litter size and pup mortality. To the best of our knowledge, our study is the first to show that maternal DMF treatment reprograms DEX+HF-induced programmed hypertension. Our results demonstrated that maternal DMF therapy activate several nutrient sensing signals—including SIRT1, AMPKα2, and PGC-1α—by which it protects adult male offspring against hypertension. This is consistent with our previous report showing that activation of the AMPK/SIRT1/PGC-1α pathway related to the reprogramming effects in another model of programmed hypertension [31,32]. These findings support the notion that the beneficial effects of Nrf2 activation on programmed hypertension may be related to activating AMPK/SIRT1/PGC-1α pathway. Interestingly, there is no synergistic effect between DEX and HF on the AMPK/SIRT1/PGC-1α pathway even though DMF rescues it. Thus, the reprograming effects of DMF might be via other metabolic and immune pathways include, but not be limited to, AMPK and SIRT1.

In support of the notion that activation of Nrf2 signaling protects against oxidative stress [15,16], our results demonstrated that maternal DMF therapy reduced oxidative DNA damage in offspring kidneys, represented by 8-OHdG immunostaining. However, 8-OHdG is just a marker of oxidative stress rather than specific mediator of hypertension development. Since hypertension is a multifactorial disorder, it remains to be determined whether oxidative stress is the major cause in the programming of offspring hypertension. Recent studies have demonstrated that inflammation and the immune system play important roles in the development of hypertension [33,34]. As DEX can affect immune system and inflammation, whether DEX-induced immune deregulation contributing to both inflammation-mediated hypertension and oxidative stress deserve further elucidation. Additionally, results of the present study suggest that DMF treatment may promote autophagy to reduce oxidative stress. Our data demonstrated that DMF restored DEX+HF-induced decreases of *Ulk1* and *Atg5* mRNA expression as well as increased the LC3-II/LC3-I ratio in offspring kidneys. A key protein in the autophagic process is LC3. The cytosolic form of LC3 (LC3-I) is conjugated to phosphatidylethanolamine to form LC3-II. Thus, the LC3-II/LC3-I ratio is a reliable marker for autophagosome formation [35]. Also, AMPK can activate ULK1 and SIRT1 can activate Atg proteins (i.e., Atg5) to promote autophagy [29]. Since DMF activates AMPK/SIRT1/PGC-1α pathway to promote autophagy, and that mitochondria are a major source of ROS, it can be presumed that selective removal of damaged mitochondria by autophagy is a protective effect of DMF in offspring kidneys against DEX+HF-induced oxidative stress damage.

In the current study, another protective effect of maternal DMF therapy on DEX+HF-induced hypertension is restoration of ADMA-NO imbalance. Oxidative stress is an oxidative shift characterized by dysregulation of NO and ROS. ADMA is considered a key player in causing a NO–ROS imbalance [24]. Conversely, reprogramming strategies targeting ADMA-related NO-ROS balance have been reported to prevent hypertension in a variety of models of developmental programming [24]. We observed that maternal DMF therapy reduced ADMA levels and increased the L-arginine-to-ADMA ratio. Given that ADMA inhibit NO production, and that NO is a known vasodilator, our data suggest that maternal DMF therapy reduces ADMA and consequently increased NO bioavailability to prevent DEX+HF-induced hypertension.

Moreover, activation of the RAS is critically linked to the development of hypertension [5,11]. We observed that renal mRNA expression of *Ren* and *Ace* were upregulated by DEX+HF exposure, while maternal DMF therapy reduced *Ren*, *Agt*, *Ace*, and *Agtr1a* expression in offspring kidney. Thus, one might expect maternal DMF therapy to block the classical ACE-Ang II-AT1R axis of the RAS in a way that opposes the development of hypertension in DEX+HF offspring rats. Since increased renal expression of tumor necrosis factor (TNF)-α was reported in DEX-treated rats [36], and since TNF-α can stimulate local ACE expression [37], it will be important that future research investigate whether RAS and inflammatory cytokines produced by the immune system closely interplay and synergistically promote elevation of BP in the DEX+HF offspring [38].

Our study has a few limitations worth noting. First, we mainly focus on the kidney in this study. The beneficial effects of DMF may be derived from other organs that regulate BP, such as the heart, the brain, and the vasculature. Another limitation is that we did not evaluate sex difference in response to DMF, as only male offspring were recruited in this study. However, the reprogrammed effects of maternal DMF therapy on male and female offspring might be different, which might deserve further clarification. Given that recent studies reported DMF may produce severe systemic side effects, in part due to non-specific *S*-alkylation of cysteine thiols [39], further studies should investigate other pro-electrophilic and non-covalent NRF2 activators to clarify their effects on Nrf2 activation in different models of programmed hypertension. Furthermore, extensive experiments in analyzing protein levels and/or functional activity of the components involved in the autophagy and RAS are required since mRNAs are not always reflected at the level of proteins and/or activities. Although the Nrf2 pathway is considered to be a master regulator of cellular oxidative stress response [15,16], it is of importance to consider it can normalize multiple factors commonly associated with hypertension, such as restoration of endothelial dysfunction [40], suppression of inflammation [41], and regulation of immune system [42]. In addition to the mechanisms we examined in the current study, further studies are needed to evaluate other factors, as outlined above, to obtain the whole picture of programming effects mediated by Nrf2 in the development of hypertension.

## 4. Materials and Methods

### 4.1. Animal Model

This study was approved by the Institutional Animal Care and Use Committee of the Kaohsiung Chang Gung Memorial Hospital (IACUC permit number: 2017071902) and carried out in strict accordance with the Guide for the Care and Use of Laboratory Animals published by the US National Institutes of Health. Virgin Sprague Dawley (SD) rats (12–16 weeks old) were obtained from BioLASCO Taiwan Co., Ltd. (Taipei, Taiwan). All rats were housed in a facility accredited by the Association for Assessment and Accreditation of Laboratory Animal Care International (AAALAC). The rats were exposed to a 12 h light/12 h dark cycle. Male SD rats were housed with individual females until mating was confirmed by the examination of a vaginal plug. Pregnant SD rats received intraperitoneal dexamethasone (DEX) (0.1 mg/kg body weight) or vehicle daily from gestational days 14 to 20 to conduct a prenatal DEX model. Male offspring rats received regular rat chow (ND; Fwusow Industry Co. Ltd., Taichung, Taiwan; 52% carbohydrates, 23.5% protein, 4.5% fat, 10% ash, and 8% fiber) or a high-fat hypercaloric diet (HF; D12331, Research Diets Inc., New Brunswick, NJ, USA; 58% fat (hydrogenated coconut oil) plus high sucrose (25% carbohydrate)) from weaning to 4 months of age, to construct the control and HF group, respectively. In addition to prenatal DEX exposure and a postnatal HF diet, rats in the DEX+HF+DMF group were born to dams received an oral dose of DMF 50 mg/kg/day (Sigma, St. Louis, MO, USA) via gastric gavage for 3 weeks during the pregnancy period. The dose of DMF used here was based on previous studies conducted with rats [18,43]. Since males are more prone to hypertension than females [44], only male offspring was selected from each litter and used in subsequent experiments. Male offspring were in five groups (*n* = 11–13 for each group): control, DEX, HF, DEX+HF, and DEX+HF+DMF.

Blood pressures were measured using noninvasive tail-cuff method (BP-2000, Visitech Systems Inc., Apex, NC, USA) as previously described [9,10]. To ensure accuracy and reproducibility, the rats were allowed to adapt to restraint and tail-cuff inflation for 1 week prior to the experiment. BP measurements were taken between 1300 and 1700 each day on a blinded basis by the same experienced research assistant. Rats were placed on specimen platform, and their tails were passed through tail cuffs and secured in place with tape. Following a 10-min warm-up period, 10 preliminary cycles of tail-cuff inflation were performed to allow the rats to adjust to the inflating cuff. For each rat, five cycles were recorded at each time point. Average of values from three stable measurements was taken. Male offspring were euthanized by an i.p. overdose of pentobarbital at 16 weeks of age. The midline of the abdomen was opened and the aorta was dissected from the adjacent vena cava, connective tissue, and fat. The aorta was cannulated with a 20- to 23-gauge butterfly, heparinized blood samples were collected, the vena cava was cut, and the kidneys were perfused with PBS until blanched. Perfused kidneys were harvested, decapsulated, divided into cortex and medulla, flash frozen in liquid nitrogen, and stored at −80 °C for further analysis.

### 4.2. Quantitative Real-Time Polymerase Chain Reaction (qPCR)

RNA was extracted from kidney cortex according to previously described methods [31]. Two-step quantitative reverse transcription PCR (qRT-PCR) was conducted using Quantitect SYBR Green PCR Reagents (Qiagen, Valencia, CA, USA) on an iCycler iQ Multi-color Real-Time PCR Detection System (Bio-Rad, Hercules, CA, USA). In addition to Nrf2, several genes related to the RAS pathway and autophagy were analyzed in this study, Components of RAS analyzed in this study included renin (*Ren*), (pro)renin receptor (*Atp6ap2*), angiotensinogen (*Agt*), angiotensin converting enzyme-1 (*Ace*), and angiotensin II type1 receptor (*Agtr1a*). Autophagy-related genes UNC-51like kinase-1 (*Ulk1*) and autophagy-related gene 5 (*Atg5*) were also analyzed. The 18S rRNA gene (*Rn18s*) was used as a reference. Primer sequences are provided in Table 3. All samples were run in duplicate. To quantify the relative gene expression, the comparative threshold cycle (*C*t) method was employed. For each sample, the average *C*t value was subtracted from the corresponding average *Rn18s* value, calculating the Δ*C*t. ΔΔ*C*t was calculated by subtracting the average control Δ*C*t value from the average experimental Δ*C*t. The fold-increase of the experimental sample relative to the control was calculated using the formula 2^−ΔΔ*C*t^.

### 4.3. High-Performance Liquid Chromatography (HPLC)

The plasma levels of components of the NO pathway, including l-arginine, l-citrulline, ADMA, and SDMA were measured using HPLC (HP series 1100; Agilent Technologies Inc., Santa Clara, CA, USA). O-phthalaldehyde/3-mercaptopropionic acid (OPA/3-MPA) was used as the derivative reagent [18]. Standards contained 1–100 mM l-arginine, 1–100 mM l-citrulline, 0.5–5 mM ADMA, and 0.5–5 mM SDMA. The recovery rate was approximately 95%.

### 4.4. Western Blotting

Western blot analysis was performed using the methods published previously [31]. Briefly, samples (200 μg of kidney cortex) were loaded on a 10–15% polyacrylamide gel and separated by electrophoresis (200 V, 90 min). Following transfer to a nitrocellulose membrane (GE Healthcare Bio-Sciences Corp., Piscataway, NJ, USA), the membranes were incubated with Ponceau S red (PonS) stain solution (Sigma-Aldrich, St. Louis, MO, USA) for 10 min on the rocker to verify equal loading. After blocking with phosphate-buffered saline–Tween containing 5% dry milk, the membranes were incubated with primary antibody. We used the following primary antibodies: a rabbit polyclonal anti-rat SIRT1 antibody (1:1000, overnight incubation; Abcam, Cambridge, MA, USA), a rabbit polyclonal anti-rat phosphorylated AMPKα2 antibody (1:1000, Santa Cruz Biotechnology, Santa Cruz, CA, USA), and a rabbit polyclonal anti-rat PGC-1α antibody (1:1000, overnight incubation; Santa Cruz Biotechnology). Following five washes with 0.1% Tween–Tris-buffered saline (TBS-T), the membranes were incubated for 1 h with horseradish-peroxidase-labeled secondary antibody diluted 1:1000 in TBS-T. Bands were visualized using SuperSignal West Pico reagent (Pierce, Rockford, IL, USA) and quantified by densitometry as integrated optical density (IOD). IOD was then normalized to total protein PonS staining. The protein abundance was represented as IOD/PonS.

### 4.5. Immunohistochemical Staining

Paraffin-embedded tissue was sectioned at 3 μm thickness. Tissue slides were deparaffinized in xylene and rehydrated in a graded ethanol series to phosphate-buffered saline. Following blocking with immunoblock (BIOTnA Biotech., Kaohsiung, Taiwan), the sections were incubated with an anti-8-hydroxydeoxyguanosine (8-OHdG) antibody (clone #N45.1, 1:100, JaICA, Shizuoka, Japan) at room temperature for 2 h. Immunoreactivity was revealed using the polymer-horseradish peroxidase (HRP) labelling kit (BIOTnA Biotech). For the substrate-chromogen reaction, 3,30-diaminobenzidine (DAB) was used. The sections were preserved under cover glass. Identical staining protocol omitting incubation with primary antibody was employed to prepare samples that were used as negative controls. All sections were stained simultaneously using the same reagents, antibody dilutions and incubation periods. Renal cells positive for 8-OHdG were examined in 10 randomly selected ×400 microscopic fields per section. The number of immunostained cells was expressed as a percentage [31].

### 4.6. Statistical Analysis

Data are reported as the mean ± standard error of mean (SEM), with statistical significance inferred where *p* < 0.05. Statistical analysis was conducted with one-way analysis of variance (ANOVA) with a Tukey post hoc test for multiple comparisons. Analyses were performed using the Statistical Package for the Social Sciences software 14.0 (SPSS, Chicago, IL, USA).

## 5. Conclusions

In summary, this is the first use of DMF therapy in pregnancy hypertension programmed by DEX+HF exposures with a focus on oxidative stress, NO, nutrient-sensing signals, autophagy, and the RAS. Our results cast a new light on applying Nrf2 activation by DMF as a reprogramming strategy to prevent the developmental programming of hypertension. Future work is certainly required to develop and translate early-life Nrf2-targeting therapies into clinical practice to reduce the global burden of hypertension-related diseases.

## Figures and Tables

**Table 1:** Morphological and biochemical values in different experimental groups.

Groups	Control	DEX	HF	DEX+HF	DEX+HF+DMF
	*n* = 11	*n* = 12	*n* = 13	*n* = 12	*n* = 13
Mortality	0%	0%	0%	0%	0%
Body weight (BW) (g)	542 ± 15	499 ± 16	531 ± 13	499 ± 12	507 ± 12
Left kidney weight (g)	1.74 ± 0.06	1.73 ± 0.06	1.69 ± 0.06	1.53 ± 0.04	1.72 ± 0.05
Left kidney weight/100g BW	0.32 ± 0.01	0.35 ± 0.01	0.32 ± 0.01	0.31 ± 0.01	0.34 ± 0.01
Systolic blood pressure (mmHg)	140 ± 3	164 ± 2 *	151 ± 2 *	170 ± 1 *^#†^	142 ± 2 ^#†‡^
Diastolic blood pressure (mmHg)	69 ± 2	79 ± 2 *	88 ± 2*	90 ± 4 *^#^	72 ± 3 ^#†‡^
Mean arterial pressure (mmHg)	92 ± 2	107 ± 2 *	109 ± 1 *	117 ± 3 *^#†^	95 ± 23 ^#†‡^

DEX = prenatal dexamethasone administration; HF = postnatal high-diet; DEX+HF = prenatal dexamethasone administration plus postnatal high-fat diet; DMF = DEX+HF group treated with dimethyl fumarate; * *p* < 0.05 vs. control; ^#^ *p* < 0.05 vs. DEX; ^†^ *p* < 0.05 HF; ^‡^ *p* < 0.05 DEX+HF.

**Table 2:** Plasma levels of NO-related parameters.

Groups	Control	DEX	HF	DEX+HF	DEX+HF+DMF
l-Citrulline (μM)	55.8 ± 5.4	56.4 ± 4.6	66.1 ± 5.9	63.4 ± 4.5	36 ± 2.1 ^#†‡^
l-Arginine (μM)	305.1 ± 27.2	348.5 ± 19.8	284.5 ± 18.1	292.9 ± 31	252.7 ± 19
ADMA (μM)	1.94 ± 0.34	1.92 ± 0.2	2.47 ± 0.22	2.27 ± 0.24	1.19 ± 0.24†‡
SDMA (μM)	1.81 ± 0.25	1.74 ± 0.15	2.05 ± 0.11	1.88 ± 0.2	1.95 ± 0.17
l-Arginine-to-ADMA ratio (μM/μM)	200 ± 40	209 ± 44	120 ± 9	144 ± 26	289 ± 65 ^†‡^

DEX = prenatal dexamethasone administration; HF = postnatal high-diet; DEX+HF = prenatal dexamethasone administration plus postnatal high-fat diet; DMF = DEX+HF group treated with dimethyl fumarate; ^#^ *p* < 0.05 vs. DEX; ^†^ *p* < 0.05 HF; ^‡^ *p* < 0.05 DEX+HF.

**Table 3:** Quantitative real-time polymerase chain reaction primers sequences.

Gene	Forward	Reverse
*Nrf2*	5 cccattgagggctgtgatct 3	5 tcagtgaaatgccggagtca 3
*Ren*	5 aacattaccagggcaactttcact 3	5 acccccttcatggtgatctg 3
*Atp6ap2*	5 gaggcagtgaccctcaacat 3	5 ccctcctcacacaacaaggt 3
*Agt*	5 gcccaggtcgcgatgat 3	5 tgtacaagatgctgagtgaggcaa 3
*Ace*	5 caccggcaaggtctgctt 3	5 cttggcatagtttcgtgaggaa 3
*Agtr1a*	5 gctgggcaacgagtttgtct 3	5 cagtccttcagctggatcttca 3
*Ulk1*	5 gagtacccgcaccagaatgt 3	5 gctgtgtagggtttccgtgt 3
*Atg5*	5 ttggcctactgttcgatcttctt 3	5 ggacagtgcagaaggtcctttt 3
*Rn18s*	5 gccgcggtaattccagctcca 3	5 cccgcccgctcccaagatc 3
